# Comparative in vitro analysis of the sliding resistance of a modern 3D-printed polymer bracket in combination with different archwire types

**DOI:** 10.1007/s00784-022-04373-5

**Published:** 2022-01-29

**Authors:** Lutz Hodecker, Christoph Bourauel, Bert Braumann, Teresa Kruse, Hildegard Christ, Sven Scharf

**Affiliations:** 1 Department of Orthodontics, University Hospital of Heidelberg, Im Neuenheimer Feld 400, 69120 Heidelberg, Germany; 2 Department of Orthodontics, University of Cologne, Faculty of Medicine and University of Cologne, Kerpener Straße 32, 50931 Cologne, Germany; 3 Department of Oral Technology, University of Bonn, Welschnonnenstraße 17, 53111 Bonn, Germany; 4 Institute of Medical Statistics and Computational Biology (ISMB), University of Cologne, Robert-Koch-Strasse 10, 50931 Cologne, Germany

**Keywords:** Sliding resistance, 3D-printed brackets, Bracket-archwire combination

## Abstract

**Objectives:**

To analyse the sliding resistance of a modern 3D-printed polymer bracket combined with different archwire types and to compare the results with conventionally used polymeric, ceramic and metal brackets. It was of further interest which bracket-archwire combination could be best qualified for clinical use.

**Materials and methods:**

The sliding behaviour was tested using an orthodontic measurement and simulation system (OMSS) for the use of two bracket types of the polymer, ceramic and metal group in combination with a 0.016 inch × 0.022 inch and 0.017 inch × 0.025 inch archwire of nickel-titanium (NiTi), titanium-molybdenum alloy (TMA) and stainless steel. Six bracket types were combined with six different archwire types and compared to each other.

**Results:**

The sliding resistance showed significant differences between various the bracket-archwire complexes. The combination of 3D-printed polymer brackets with both steel archwire cross-sections showed the least values of sliding resistance (average 23–29%), while the combination of ceramic brackets with TMA archwires presented the highest (average 47%).

**Conclusions:**

The present study could show that modern 3D-printed bracket materials can have similar or even better mechanical properties than conventional ones regarding sliding resistance. Although the combination of bracket and archwire material is decisive for low sliding resistance values, the selection of the bracket material seems to have a greater influence than the selection of the archwire material or its cross section.

**Clinical relevance:**

It might be possible in future to combine aesthetic and biomechanical requirements for aesthetic brackets by using 3D-printing technology.

## Introduction

There are different bracket systems available for the orthodontic treatment with fixed bracket appliances. These are mainly preprogrammed straight-wire brackets made of metal or ceramic which differ e.g. in the ligating method, the material composition or the biomechanical properties. The majority of patients wish to wear aesthetically pleasing bracket materials which help them to feel more comfortable with their appliance or to accept their treatment time. For this reason, many patients wish to be treated with polymer or ceramic brackets. Numerous studies were found in literature which showed that metal brackets offer superior biomechanical properties than ceramic brackets, especially regarding sliding behaviour [1–3]. Other studies could demonstrate that lining the ceramic bracket slot with stainless steel, glass or gold materials can reduce the high sliding resistance of ceramic brackets [4]. The aesthetic polymer materials used so far were mainly made of polycarbonate or polyurethane [5]. Despite their aesthetic advantages, they have not established themselves for routine clinical practice. The problems were related to discoloration, lack of strength and stiffness, binding wing fractures and torque loss [6]. To compensate these deficits, polymer brackets were also developed with different filler materials, e.g. ceramic or fiberglass, or reinforced with metal slots [7]. All these efforts demonstrate that in the majority of cases the orthodontist is faced with the challenge to decide between the biomechanical properties or the patients’ aesthetic demands when choosing a bracket system. For this reason, it would be desirable to use a bracket material that combines both, the biomechanical and aesthetic requirements. In addition, it would be a further advantage of future bracket systems to turn away from conventional, standardized bracket designs towards a patient-specific, customized bracket system. Modern CAD/CAM technologies could satisfy these requirements in order to increase the treatment efficiency. A completely digital workflow, as presented by Krey et. al., could take into account the patients tooth shapes, sizes or position [8]. However, a completely digital workflow not only means the intraoral scanning of the dental arches, the digital positioning of conventional brackets or the use of 3D-printed bonding trays, but also the fabrication of the brackets themselves using 3D-printing technology. There is currently a great need for research in this field in order to develop a customized bracket system with an appropriate bracket material. The precondition for the successful use of a 3D-printed, customized bracket system is a printable polymer material with biomechanical properties comparable to those of conventional bracket materials.

**Fig. 1:**
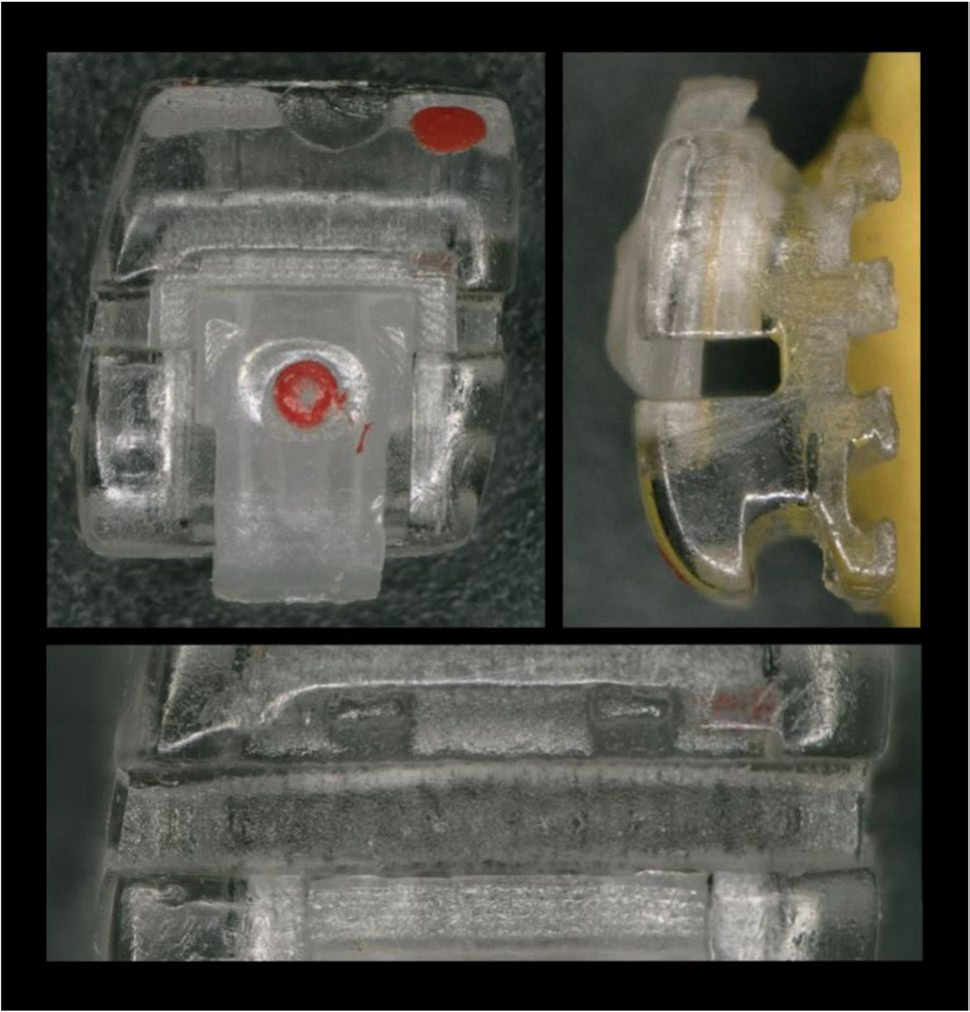
Detailed view of the modern polymer 3D-printed Shark SL bracket from vestibular and mesial direction

Thus, this study aimed to analyse the sliding behaviour of a modern 3D-printed polymer bracket in combination with various archwire types and to compare the results with commonly used materials. Within this investigation the self-ligating Shark SL polymer bracket was analysed (Fig. 1). This bracket consists of a polymer filled with polycrystalline alumina ceramic and was produced using a Digital Light Processing (DLP) 3D-printer.

## Materials and methods

Two bracket types from three different material groups (polymer, ceramic and metal) were selected for this investigation. The selection of the bracket types, as well as the experimental setup for sliding resistance measurements, was similar to another study presented by the same author group where only one archwire type was used [9]. All brackets were for tooth 23 with the similar slot size type (0.018 inch × 0.025 inch) and torque value (0°). As a representative for the polymer group the Brillant® bracket (injection moulded polyoxymethylene, Forestadent Bernhard Förster GmbH, Pforzheim, Germany) as well as the 3D-printed self-ligating Shark SL bracket (Dentalline GmbH & Co. KG, Birkenfeld, Germany), for the ceramic group the discovery® pearl (Dentaurum GmbH & Co. KG, Ispringen, Germany) and the Inspire Ice™ bracket (Ormco Europe, BR Amersfoort, The Netherlands) and for the metal group the discovery® and equilibrium® ti bracket (Dentaurum GmbH & Co. KG, Ispringen, Germany) were investigated.

**Fig. 2:**
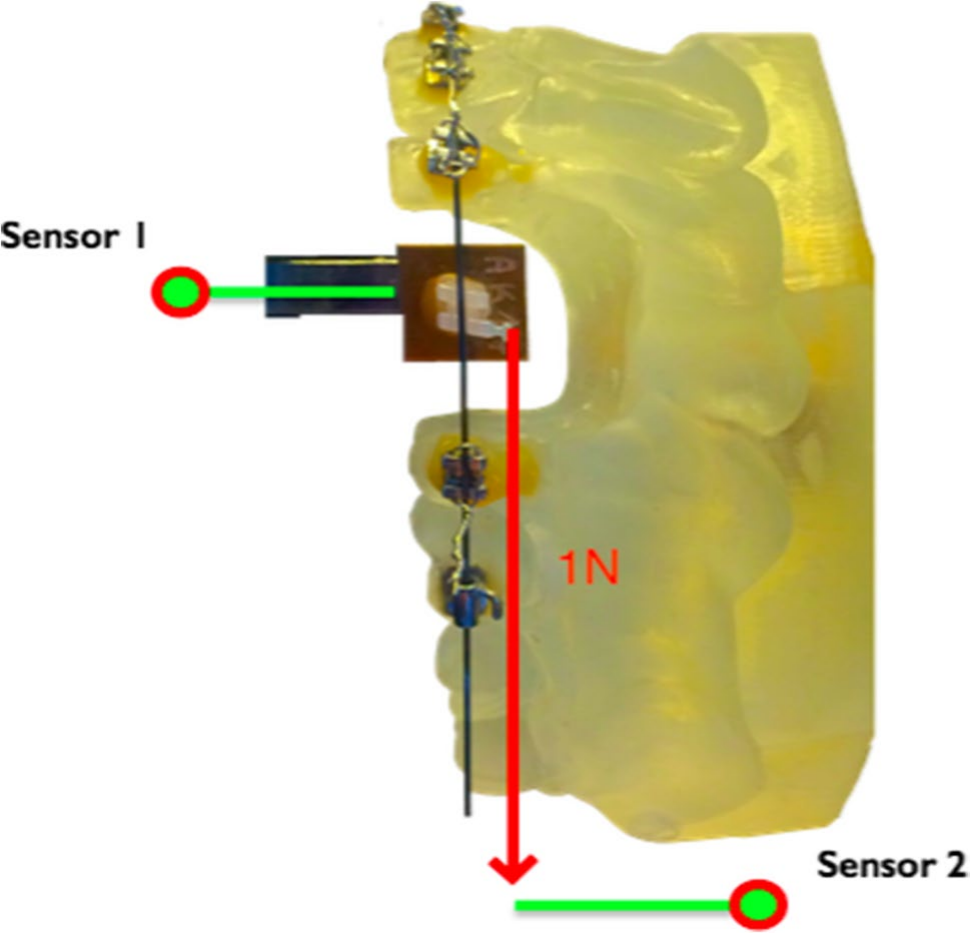
Schematic illustration of the experimental setup of the orthodontic measurement and simulation system (OMSS) [9]

The force losses due to sliding resistance were measured with the help of an orthodontic measurement and simulation system (OMSS, Fig. 2). This is an apparatus that simulates an orthodontic tooth movement after applying a specific orthodontic force [10]. It records the occurring forces of the test brackets three-dimensionally via force/torque sensors. A resin replica of an upper jaw model by Frasaco (Frasaco GmbH, Tettnang, Germany) was used for fixing the archwires, in which tooth 23 was replaced by a test bracket. The tooth 24 also had to be removed to ensure a distalisation path. The model and brackets were mounted in the OMSS such a way that initially no forces were measurable. Only then was the experimental force applied to the test bracket. The bracket was linked via an arm structure to the first sensor of the OMSS for measuring the occurring forces. A second sensor was used to measure the applied force level, implemented by connecting a nickel-titanium spring coil (rematitan®LITE; Dentaurum GmbH & Co. KG, Ispringen, Germany) to both, the first sensor (via the hook of the experimental bracket 23) and the second sensor. When simulating an orthodontic tooth movement, the resulting resistance due to force loss was calculated by subtracting the force level at the bracket sensor from the orthodontic force applied. In this example, a distalizing force of 1 N was applied with the help of the nickel-titanium spring coil. Each bracket tested was uniformly ligated according to the recommendations of Schumacher et al. [11]. They figured out that the ligature process has a significant influence on the friction behaviour between bracket and archwire. Therefore, the ligature (remanium® preformed ligature 0.010 inch; Dentaurum GmbH & Co. KG, Ispringen, Germany) was closed and then reopened with a 180° turn. The closing mechanism of the self-ligating Shark SL bracket was permanently blocked in the opened position so that this bracket type could be ligated conventionally for all experiments. Following these preconditions, the measurements started when distalizing the test bracket, which means that the test bracket combined with the first sensor was moved towards the second sensor by spring force. In the context of this distalization path, 200 measured values of force loss were recorded and noticed in a therefore developed software as well as in the program Microsoft Excel (Microsoft Corporation, Redmond, WA, USA) for further analyses [9]. All bracket types were measured with five samples combined with 6 different archwire types. They differed in cross-section and material. The archwire materials used were remanium® (spring hardened stainless steel), rematitan® sl (superelastic nickel-titanium alloy, NiTi) and rematitan® SPECIAL (titanium-molybdenum alloy, TMA), each in the dimensions 0.016 inch × 0.022 inch and 0.017 inch × 0.025 inch (all wires from Dentaurum GmbH & Co. KG, Ispringen, Germany).

### Statistical analysis

Based on the 200 individual measured force loss values of each of the five test brackets, mean and standard deviation were calculated for all tested bracket-archwire combinations to obtain a single force loss value of every test bracket. Each group consisted of 5 sample brackets for which the median, the mean and the standard deviation were calculated. The median values were used for the statistical tests. Because of the fact that a normal distribution of the results cannot be assumed for a sample size of 5, non-parametric statistical tests were used, so the Kruskal–Wallis H Test followed by the Mann–Whitney U test pointed out statistically relevant significances between the different groups. A significance level of 0.05 was defined for all evaluations as statistically significant. The statistical evaluation was undertaken with the Statistical Package for Social Sciences, version 25.0 (IBM, Armonk, New York, USA).

## Results

**Fig. 3:**
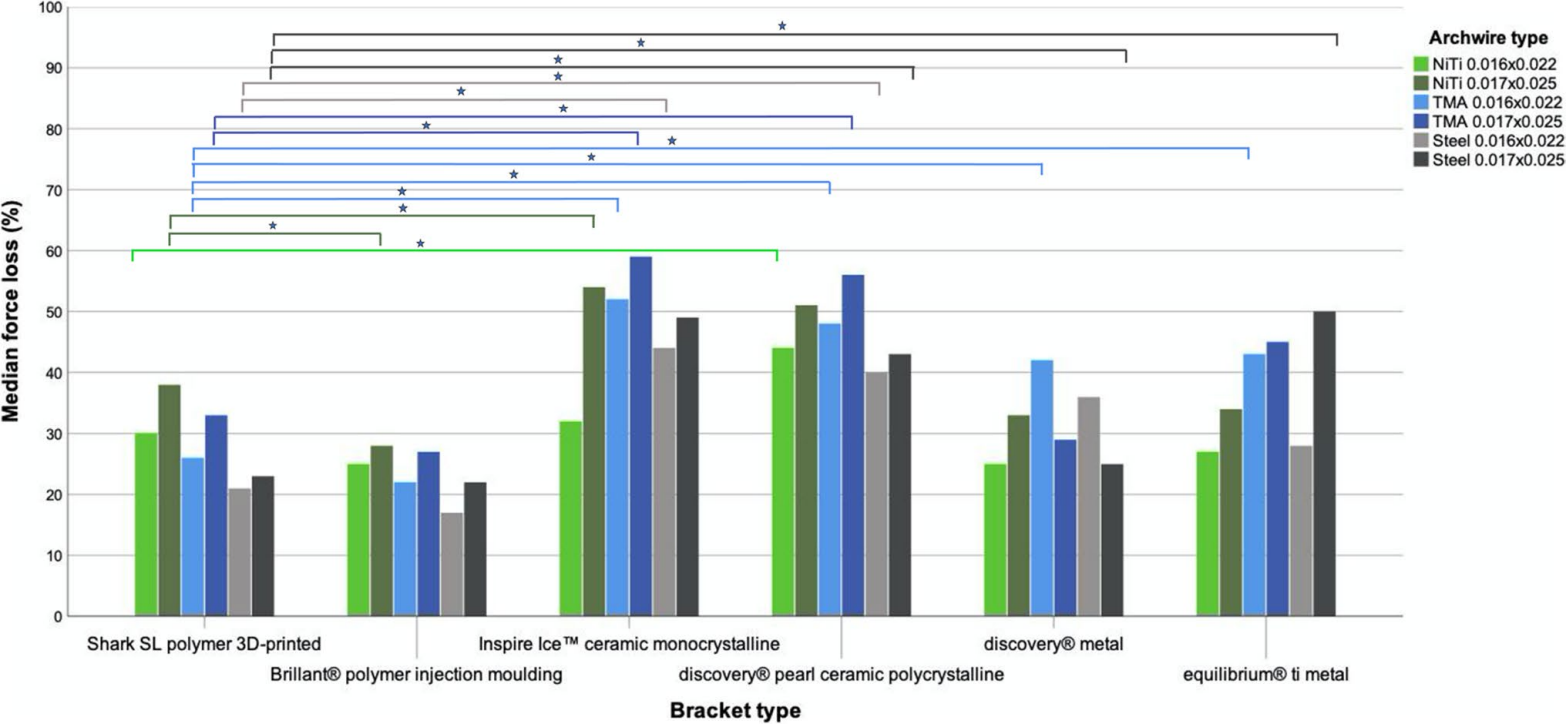
Visual representation of force losses due to sliding resistance of all bracket-archwire combinations. The significances were only illustrated combined with the 3D-printed Shark SL bracket. The stars represent statistical significance (*p* ≤ 0.05)

In some cases, significant differences in measured force loss values due to sliding resistance could be observed between the different bracket types (Fig. 3 and Tables 1, 2, 3).

**Table 1:** Measurements of the force losses of all bracket types in combination with the nickel-titanium (NiTi) archwires. (*SD* standard deviation)

	Force loss due to sliding resistance [%]									
	NiTi					NiTi				
	0.016 inch × 0.022 inch					0.017 inch × 0.025 inch				
	25%	Median	75%	Mean	*SD*	25%	Median	75%	Mean	*SD*
Bracket type										
Shark SL	26	30	39.5	32.2	7.6	31	38	43	37.2	6.4
Brillant®	14	25	28.5	24	5.1	22	28	29.5	26.2	4.1
Inspire Ice™	27.5	32	43	34.6	10.8	46	54	56.5	51.8	6.7
discovery® pearl	38	44	59	47.6	13.7	34	51	55	45.8	11.5
discovery®	16.5	25	35	25.6	9.6	25.5	33	38.5	32.2	7.1
equilibrium® ti	24.5	27	29.5	27	2.7	23	34	38	31.2	9.2

**Table 2:** Measurements of the force losses of all bracket types in combination with the titanium-molybdenum alloy (TMA) archwires. (*SD* standard deviation)

	Force loss due to sliding resistance [%]									
	TMA					TMA				
	0.016 inch × 0.022 inch					0.017 inch × 0.025 inch				
	25%	Median	75%	Mean	*SD*	25%	Median	75%	Mean	*SD*
Bracket type										
Shark SL	20	26	32	26	7.3	25	33	36	31	6.7
Brillant®	18	22	26	22	4.7	23	27	30.5	26.8	4.1
Inspire Ice™										
	40.5	52	81	59	21.7	48.5	59	75	61.2	16.2
discovery® pearl	43	48	60.5	51	9.1	51.5	56	58.5	55.2	3.6
discovery®	33.5	42	46	40.2	6.6	25	29	37.5	30.8	7.7
equilibrium® ti	33.5	43	53.5	43.4	11.4	30.5	45	46	39.6	11.1

**Table 3:** Measurements of the force losses of all bracket types in combination with stainless steel archwires. (*SD* standard deviation)

	Force loss due to sliding resistance [%]									
	Stainless steelx					Stainless steel				
	0.017 inch × 0.025 inch					0.017 inch × 0.025 inch				
	25%	Median	75%	Mean	*SD*	25%	Median	75%	Mean	*SD*
Bracket type										
Shark SL	12.5	21	23	18.4	5.6	20.5	23	26.5	23.4	3.6
Brillant®	7.5	17	20	14.4	6.8	14.5	22	27	21	7.1
Inspire Ice™	33.5	44	56	44.6	13.1	38	49	84.9	58.8	26.2
discovery® pearl	36.5	40	49	42.2	6.8	38	43	47.5	42.8	5.1
discovery®	33.5	36	39	36.2	3.3	20	25	42.5	30	13.4
equilibrium® ti	22	28	54	36	16.6	36	50	55.5	46.6	13.2

The comparison of the force loss of the polymer bracket group with them of the ceramic and metal group revealed the smallest values for all bracket-archwire combinations. Within this group, the least values were found for the Brilliant® bracket (average 23%), closely followed by the Shark SL bracket (average 29%). The ceramic bracket group demonstrated the highest friction values (average 47%), the metal group ranges between both groups (average 31 and 37%). Regarding the polymer bracket-archwire combinations, it can be stated that the NiTi archwires showed the highest sliding resistance, followed by the TMA and stainless steel archwires with the smallest values. The increase in cross-section from 0.016 inch × 0.022 inch to 0.017 inch × 0.025 inch led to an increase in sliding resistance of 21% for NiTi and TMA archwires and only 9% for stainless steel archwires. Furthermore, the combination of stainless steel archwires with the polymer brackets showed the least force loss values of all combinations.

When regarding the ceramic group, the use of TMA archwires revealed the highest sliding resistance, especially for the Inspire Ice™ bracket (52 to nearly 59%). An increase in cross-section resulted in an increase in force loss, most obvious found for the Inspire Ice™ bracket with the NiTi archwire (32 to 54%). The least force loss with the polycrystalline discovery® pearl bracket was found for the combination with both steel archwire cross-sections.

The metal group showed less force loss values than the ceramic group but higher values than the polymer group. An increase in cross-section led to an increase in sliding resistance for all arch wire combinations, with two exceptions. Within this investigation, the combination of the discovery® metal bracket with the TMA and steel archwires decreased the force loss when increasing the cross-section.

## Discussion

It was the intention of this study to evaluate the biomechanical properties of a modern 3D-printed polymer bracket material in combination with six different archwire types and to compare them with the properties of conventional, commonly used ones. The 3D-printing technology represents a promising future manufacturing method for orthodontic brackets, because it could fulfil many requirements for an ideal orthodontic bracket. Integrated into a completely digital workflow, as presented by Krey or Panayi, 3D-printed brackets could take into account patient-specific features such as tooth shape, size or colour [8, 12]. Completely individualized fixed appliances could be produced in future using 3D-printing technology. It is a precondition for this procedure that printed bracket materials have not only the necessary aesthetic but also the biomechanical properties. The biomechanical properties of an ideal orthodontic bracket include less force loss due to sliding resistance and sufficient torque capacity.

The problem of force losses during arch-guided tooth movement has been discussed extensively in the literature. Burrow stated that total resistance composes of static and kinetic friction [13]. Static friction opposes the applied force and has to be overcome to start a movement. Kinetic friction opposes the direction of movement of the object and is usually less than static friction. In orthodontic movements kinetic friction is irrelevant. It represents a rather quasi-static dynamic process because the teeth are alternately tipped and straightened up again along the archwire [13]. Friction is only a small part of resistance to movement and occurs from the interaction of an archwire with the bracket or ligature. Kusy and Whitley divided this resistance into three parts: friction, as mentioned before, binding, created by the contact of the archwire with the corner of the bracket and notching, if a permanent deformation of the archwire occurs due to interaction with the bracket [14].

There is a large number of studies concerning sliding resistance tests in literature. It should be mentioned that the results of sliding behaviour determined by the OMSS and presented here are from an in vitro study. This type of experiments does not consider the conditions in the oral cavity, such as body temperature, saliva or calculus. Nevertheless, many researchers have investigated the influence of the ligature system, i.e. the difference between self-ligating and conventionally ligated brackets [15]. Others studied the difference between ceramic and metal brackets [4, 16–18]. Only a few examined all three material groups under the same experimental conditions. Within this investigation, it was found that the polymer brackets showed the least force loss values followed by steel, titanium and ceramic brackets, almost for all bracket-archwire combinations. Similar results could be detected by other investigators [3, 19]. Here, the ceramic brackets revealed the highest force loss values and, within this group, the monocrystalline bracket showed slightly higher values than the polycrystalline ones, especially combined with the 0.017 inch × 0.025 inch cross-sections. These findings have also been described in the literature before [2, 20]. For this reason, there have been many attempts to reduce these high force loss levels of ceramic brackets in the past, e.g. by the incorporation of a metal, silica, glazed or zirconia-based slot [20–24]. The good sliding properties of polymer materials have also been used to coat guiding archwires in order to reduce the sliding resistance [25, 26].

According to the findings of the present study, 3D-printed bracket materials could provide a promising alternative bracket material to ceramic or metal materials. The polymer bracket group showed the least force loss values especially in combination with stainless steel archwires of both cross-sections. The combination of the 0.016 inch × 0.022 inch stainless steel archwire with the 3D-printed Shark SL bracket revealed approximately 42% less force loss than the combination with the steel discovery® bracket whereas the latter combination is considered as ßgold standard for archwire guided tooth movement by many practitioners. According to other studies, the greatest force loss values were found for the combination of the 0.017 inch × 0.025 inch TMA and NiTi archwire with ceramic brackets [27]. In addition, it was found that an increase in cross-section also led to an increase in force loss for all bracket-archwire combinations, except the step from 0.016 inch × 0.022 inch to 0.017 inch × 0.025 inch stainless steel and NiTi archwire combined with the discovery® steel bracket. All these findings allow the conclusion that the extent of force loss is more influenced by the choice of the bracket material than by the choice of the archwire type.

It has to be mentioned that sliding resistance also depends on the slot dimension and thus on the manufacturing precision. This correlation becomes even more important when comparing brackets from different manufacturers. To assess the accuracy or discrepancy of the slot and archwire size, the torque play of the bracket slot can be used as a reference [28]. Earlier studies found out that the actual torque play often differs from the theoretical one due to oversized bracket slots [29]. Joch et al. conclude from their investigation that the accuracy of the manufacturer slot dimension should not be taken for granted [28]. In order to solve the problem of varying manufacture-related precision, a DIN standard for brackets and tubes (DIN 13,971–2 [30]) was introduced that regulates the nominal dimensions of orthodontic brackets and tubes within their tolerance limits [28]. Several investigators have found deviations from the nominal bracket slot dimension within their studies [31–33]. There are assumptions that some manufactures deliberately produce their brackets close to the upper DIN limit in order to simplify the treatment for the orthodontists or to suggest them a well-working bracket-archwire system. This approach reduces sliding resistance but also increases tooth tipping and torque play, which also leads to side effects that must be corrected by the orthodontist. Unfortunately, the archwires also show inaccuracies and deviations von DIN norm (DIN 13,971 [34]). Joch et al. figured out that two-thirds of their examined archwire types exceeded the limit for height and one-third the limit for width [28]. These circumstances demonstrate that in the majority of cases orthodontists have to treat their patients with conventionally produced brackets and archwires of varying and to be honest of unknown precision.

Within the context of the experiments presented here, all samples of each brackets type were measured with the same archwire, so that manufacturer-related variances of the archwires could be neglected when comparing different brackets types. Here, the Shark SL bracket showed less force loss levels in combination with the 0.017 inch × 0.025 inch stainless steel archwire than the discovery® bracket combined with both stainless steel archwires. Considering the slot precision determined from an earlier work, the Shark SL bracket showed an even more precise slot than the discovery® bracket [9]. The Brilliant® and Inspire Ice™ bracket showed the most accurate slots, closest to the DIN norm of 457 µm, followed by the Shark SL, discovery® pearl, discovery® and equilibrium® ti bracket. The high slot precision of the Inspire Ice™ brackets could be an additional reason for the high force loss values.

**Fig. 4:**
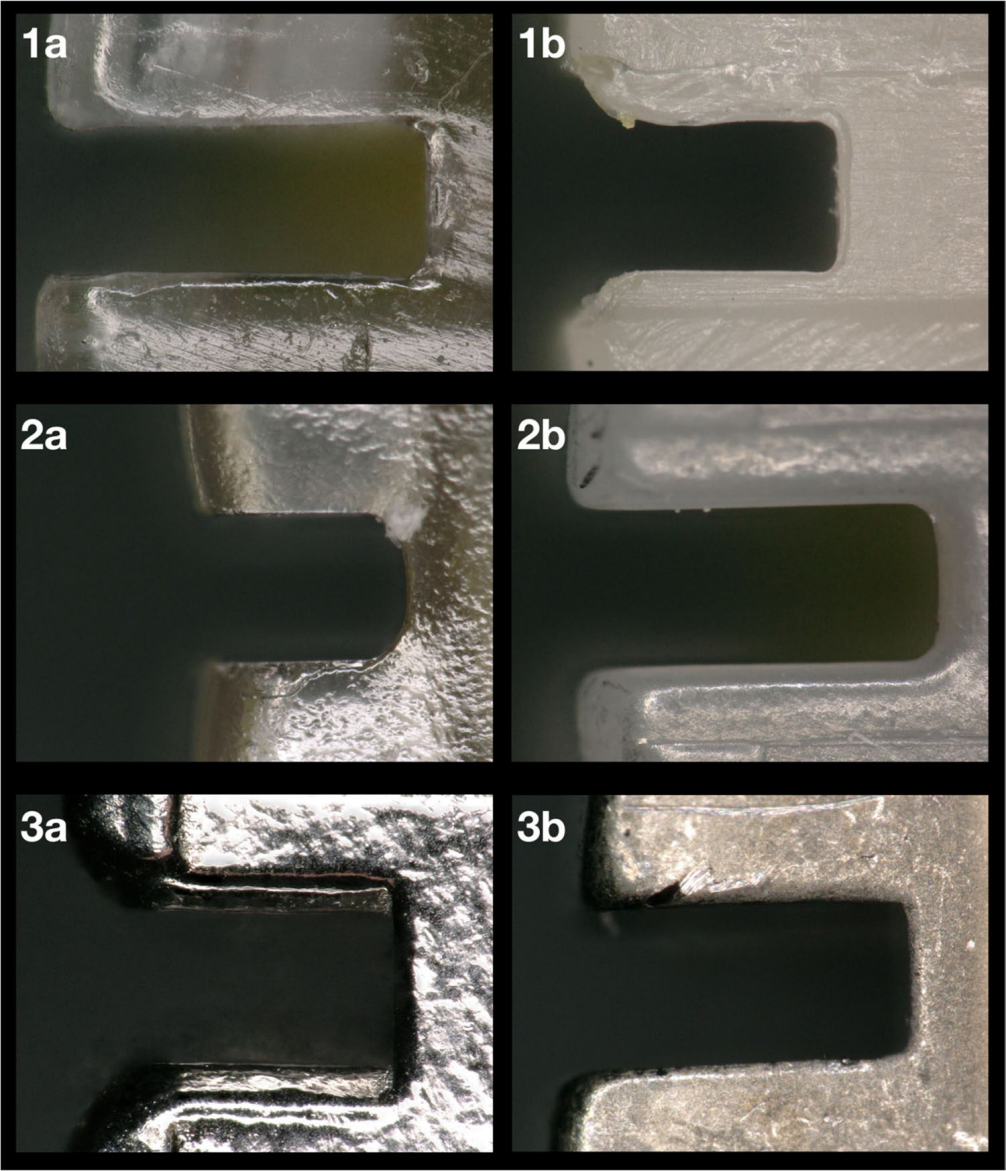
Mesial slot areas and bevel designs of all tested bracket types. 3D-printed Shark SL (1a) and Brillant® bracket (1b), the ceramic group: Inspire Ice™ (2a) and discovery® pearl bracket (2b) and the metal group: discovery® (3a) and equilibrium® ti bracket (3b). Different bevel designs and slot sizes could be observed (light microscope image, × 200 magnification)

In addition to bracket and archwire precision, the slot design also influences the force loss due to sliding resistance [35]. Earlier studies concluded that increasing the bevel angle reduces binding scratches on the archwire and thus the force loss due to sliding resistance [36]. Figure 4 shows the bevel designs of the investigated brackets. It can be identified that the Inspire Ice™ bracket illustrates the least bevel angle of all brackets examined. In addition, most chipping effects were found with this bracket type [9]. The correlation between bevel angle und force loss could be confirmed with the results found here. The Inspire Ice™ bracket inhibited the highest force loss levels, especially in combination with the TMA archwires which are probably due to explicit binding and notching effects.

Besides sliding properties and slot precision, a sufficient torque capacity and colour stability are crucial for the clinical use of polymer brackets. Möller et al. tested the torque stability of polycarbonate and polyurethane brackets filled with ceramic or fiberglass as well as reinforced ones with metal slots. They found out that only the reinforcement with metal slots led to significantly less slot deformation and higher torque values and suggested only them for clinical use [37]. Future research should verify the torque capacity of 3D-printed bracket materials. It is not yet determined whether their torque stability is comparable to that of ceramic or metal brackets, but in addition, it should be mentioned that it should not be the material properties of metal brackets but rather the physiological cellular processes of the parodontium that determine the effectiveness for torque capacity. Too high torque values, as could possibly be applied by metal brackets could cause undesirable side effect, i.e. root resorption. 3D-printed polymer brackets only have to apply as much torque capacity as is necessary for physiological torque movements.

Besides torque capacity, 3D-printed bracket materials should also be characterized by colour stability. This stability depends largely on the type and composition of the polymeric resin used. Initial studies of currently available resins show that the staining effect of wine in particular or changes in colour with aging still require further developments [38].

## Conclusions

This study could demonstrate that modern 3D-printed polymer bracket materials can provide comparable or even better biomechanical properties than conventional ones regarding sliding resistance. It was found that polymer brackets showed the best sliding properties not only in combination with steel archwires but also with NiTi and TMA archwires for both cross sections. This suggests that the choice of bracket material has a bigger influence on the sliding properties than the choice of archwire material.

However, materials research is still necessary to investigate fracture stability, torque capability and colour stability. If these material-specific properties satisfy the requirements of a sufficient tooth movement, the orthodontists could in future be able to produce customized bracket systems by themselves using CAD/CAM processes. This could lead to patient-specific, individually programmed bracket systems, which could increase treatment efficiency and be more in line with the straight wire concept than conventionally produced bracket systems.
